# The Standpoint of the Other Fellow

**Published:** 1899-02

**Authors:** 


THE STANDPOINT OF THE OTHER FELLOW.—Hazlett, the English historian, in his "Life of Napoleon," in speaking of Bonaparte's restoration of ecclesiastical power in France after its abrogation by the Revolution, says: "Every man is ready to condemn wrong doing until it comes his time to do wrong."

Now, from the standpoint of the medical practitioner, the general practitioner who has to "go," rain or shine, blow, snow or freeze, night or day, sick or well, convenient or inconvenient; refusing no calls—not even those from known "dead beats," because it may be a child that is sick, or a woman, you know,—there is no greater wrong, none to be more generally condemned, than the failure, neglect or refusal to pay his modest little bill by those who are *able to pay.* In this we agree with the general practitioner. We haven't heard from the other fellow; how do you know he's "able

*Canned foods are said to have been found at Pompeii, sound, after eighteen hundred years.

to pay?" Well, we'll suppose he's able to pay. He is justly to be censured if he can pay and won't pay the good doctor. We published a case last month of a mean fellow who wouldn't pay the doctor for his baby. Now, it's all right to censure these fellows for "wrong doing." But, doctor, you who read this, haven't you a beam in your eye, yet see the mote in that of the other fellow? Is it your time to "do wrong"? Don't you owe anybody for a "baby,"—a Red Back baby "brought forth" after much "labor" (and expense) month after month, and year after year, and sent to you in a neat wrapper, and spick and span? How does your subscription account stand with the TEXAS MEDICAL JOURNAL? You ought to know; a statement has been sent you four times a year, if you are one of the fellows we are poking at. If it is wrong to not pay the doctor, and we agree with you, is it not wrong for the doctor to not pay for the JOURNAL? Read those "Dont's" in another column, and hurry to the postoffice and clear your conscience; *then* you can pitch into your fellow, and we'll pat you on the back and say, "hit him again."

